# Biomechanical effect of cage size in single-level anterior cervical discectomy and fusion: a finite element analysis

**DOI:** 10.1186/s12891-025-08850-2

**Published:** 2025-07-04

**Authors:** Qianqian Zhang, Yusheng Jin, Liming He, Kun Zhang, Lingfeng Chen, Weiyi Chen, Haoyu Feng

**Affiliations:** 1 Third Hospital of Shanxi Medical University, Shanxi Bethune Hospital, Shanxi Academy of Medical Sciences, Tongji Shanxi Hospital, Taiyuan, 030032 China; 2 College of Biomedical Engineering Taiyuan University of Technology, Taiyuan, 030024 China; 3 School of Automation and Software Engineering Shanxi University, Taiyuan, 030031 China

**Keywords:** Finite element analysis, Cage size, Anterior cervical discectomy and fusion, Cervical stability, Range of motion, Facet joint pressure, Intervertebral disc pressure, Complete fusion

## Abstract

**Background:**

Anterior cervical discectomy and fusion (ACDF) is a common surgical procedure for treating cervical spine diseases, but its anterior approach can lead to complications such as dysphagia and carotid artery injury due to the large incision. However, performing ACDF under a percutaneous endoscopic approach can effectively mitigate these issues. Considering the need for smaller-sized cages in endoscopic procedures, this study explores the feasibility of using small-sized cages for percutaneous endoscopic ACDF surgery.

**Methods:**

The finite element method is used in this paper to construct cervical spine surgical models with three different sizes of cages implanted, studying the impact of size on cervical biomechanical performance. The dimensions of the cages remain constant in length and height, with a length of 14 mm and a height of 6 mm, and widths of 7 mm, 10 mm, and 14 mm, respectively.

**Results:**

In a complete fusion state, the range of motion of the surgery level decreased, while adjacent segments showed a compensatory increase in range of motion. Intervertebral disc pressure increased in adjacent discs during flexion and extension. Facet joint pressure in the operated segments generally decreased across all conditions compared to the intact model, but in non-surgical segments exhibited varied compensatory increases under different conditions. Smaller cages led to increased von Mises stress on the cage and endplates, with stress distribution varying by motion condition.

**Conclusion:**

The results show that, using a 10 mm wide polyetheretherketone cage in complete fusion does not significantly affect postoperative vertebral stability or adjacent segment degeneration risk. Additionally, the risk of subsidence is relatively low, making it a suitable cage option for percutaneous endoscopic ACDF surgery.

## Background

Cervical spondylosis is a common condition that increases with aging, primarily caused by intervertebral disc degeneration [1, 2]. The pathological discs protrude and compress surrounding cervical spinal cord or nerves, manifesting as neck pain, radiating pain in the upper limbs and reduced mobility. Conservative treatments like medication [3], traction [4], and massage [5] can alleviate symptoms to some extent. However, surgical intervention becomes crucial when the condition worsens significantly, impacting the patient’s daily life [6].

Since the 1950 s, ACDF, as a standard technique, has been widely used in patients with cervical spondylosis [7, 8]. ACDF involves the removal of the degenerated disc and the insertion of an interbody fusion system, not only achieving decompression of the compressed nerves or spinal cord but also ensuring postoperative cervical stability. However, the anterior approach can lead to temporary or permanent complications, such as dysphagia, recurrent laryngeal nerve injury, carotid artery injury, esophageal injury, and even pharyngoesophageal diverticulum [9–11]. With medical advancements, more surgeons are opting for minimally invasive surgical techniques for cervical spondylosis [12]. Percutaneous endoscopic cervical discectomy (PECD) involves removing degenerated intervertebral discs through an endoscopic surgical approach alleviating the impact of the diseased discs on surrounding tissue structures [13, 14]. Compared to ACDF, PECD has smaller incisions, effectively addresses postoperative complications associated with open surgery, significantly reduces intraoperative bleeding, lowers infection risks, shortens the treatment period, and hastens patient recovery. However, since PECD only removes part of the diseased intervertebral discs and has limited decompression capability, it is not suitable for patients with severe disc degeneration [10, 15, 16].

To avoid postoperative complications of open surgery and expand the range of surgical treatment to cover most cervical spondylosis conditions, we propose an optimized surgical strategy of ACDF via an endoscopic surgical approach. Due to the limited diameter of the endoscopic surgical approach [12, 17], a smaller-sized cage is necessary to ensure successful surgery. The cage plays an important role in restoring intervertebral height, postoperative healing, and maintaining vertebral stability [18, 19]. However, reducing the size of the cage can decrease the contact area and grafting window, which may increase the risk of subsidence, lower fusion rates, and undermine postoperative vertebral stability [20, 21]. Therefore, studying the impact of cage size on the biomechanical performance of the cervical spine after surgery is of great significance for achieving ACDF via an endoscopic surgical approach.

Finite element (FE) studies, being cost-effective compared to clinical and vitro experiments and less influenced by surgical conditions and patient variability, are particularly suitable for preliminary research on the impact of smaller cages on cervical biomechanics [22]. This study aims to design a cage that can be inserted through an endoscopic surgical approach for minimally invasive surgery, thereby reducing patient trauma. By constructing a finite element model of the healthy human C3-C7 cervical spine segments and simulating ACDF surgery with cages of widths 14 mm, 10 mm, and 7 mm (height 6 mm, length 14 mm), we compare the mechanical parameters of the cervical spine model before and after surgery. This analysis assesses the impact of smaller-sized cages on postoperative cervical biomechanics and evaluates their feasibility in minimally invasive ACDF surgery.

## Methods

### Development of an intact model

**Fig. 1:**
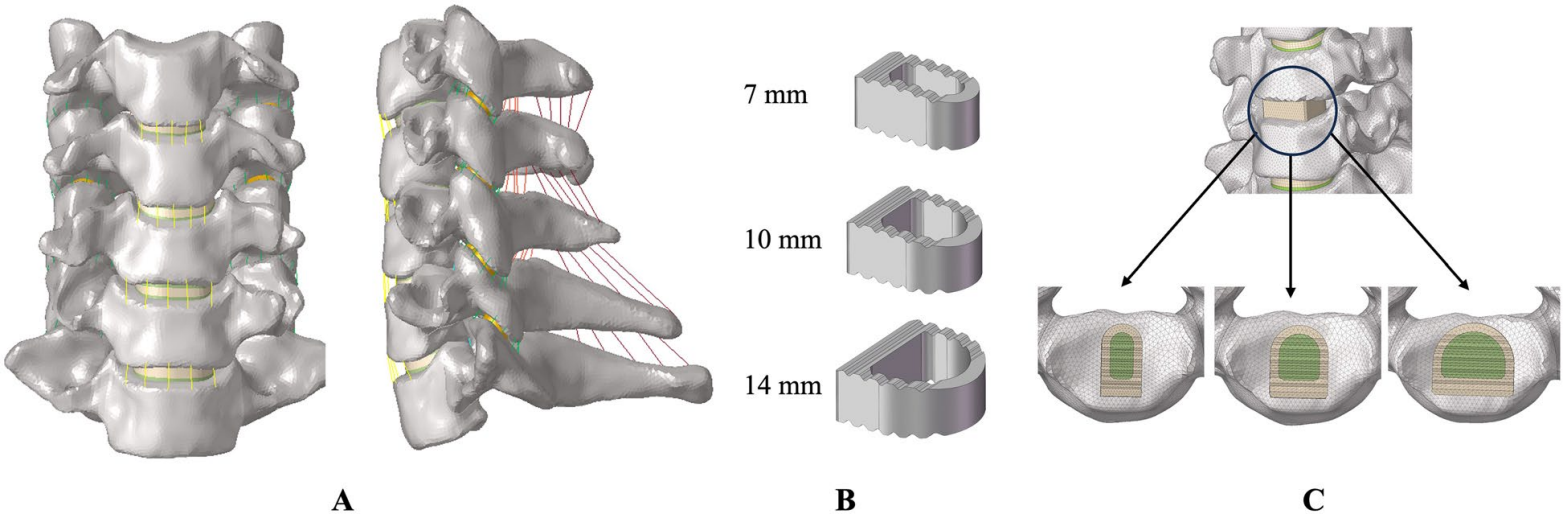
Finite element model of the C3–C7 cervical spine. **A** front view and sagittal view of the intact model; **B** cages with widths of 7 mm, 10 mm, and 12 mm; **C** three cage models assembled in ACDF models

The cervical spine models were constructed based on computed tomography (CT) scans of a healthy adult. The volunteer, a 37-year-old male with a height of 170 cm and weight of 67 kg, had no history of cervical spine diseases. Mimics software (Materialise Inc., Leuven, Belgium) was utilized to extract CT information and perform the initial reconstruction of a 3D model of the cervical spine. Subsequently, Geomagic Wrap software (Geomagic, Inc., Research Triangle Park, NC, USA) was employed for denoising, smoothing, and creating a cancellous bone model of the cervical spine. Following the anatomical structure of the human body, intervertebral discs and facet cartilage were modeled and filled into the spaces between vertebrae and joints using Solidworks software (Waltham, Massachusetts. USA). Hypermesh software (Altair Engineering, Inc. Executive Park, CA, USA) was then used to mesh the established cervical spine model, assign material properties, and construct cervical ligaments. As shown in Table 1 and Fig. 1. The mesh model was imported into Abaqus (SIMULIA Inc.). The contact settings between the intervertebral discs and vertebra, as well as between the cartilage and vertebra, were established as Tie, while connecting cancellous bone, cortical bone, and posterior structures through shared nodes. The vertebral body consisted of cancellous bone, cortical bone, and posterior structures, partitioned using tetrahedral mesh elements, with a 0.5 mm thick cortical bone. The intervertebral disc was composed of endplates, annulus fibrosus, and nucleus pulposus, meshed using hexahedral elements, with a 0.5 mm thick endplate. Ligaments included anterior longitudinal ligament (ALL), posterior longitudinal ligament (PLL), ligamentum flavum (LF), capsular ligament (CL), and interspinous ligament (IL), simulated using tension-only truss elements. Facet joints were meshed using tetrahedral elements.

**Table 1:** Material properties and mesh types of the cervical finite element model

Description	Young’s Modulus (MPa)	Poisson’s Ratio	Element type	Cross-section (mm^2^)
Cancellous bone	450	0.25	C3D4	—
Cortical bone	10,000	0.3	C3D4	—
Posterior	3500	0.25	C3D4	—
Annulus fibers	4.2	0.4	C3D8H	—
Nucleus pulposus	1	0.49	C3D8H	—
Facet joint	10	0.3	C3D4	—
ALL	26.3	0.4	T3D2	11
PLL	22.2	0.4	T3D2	10.4
LF	3.1	0.4	T3D2	50.1
CL	3.3	0.4	T3D2	46.6
IL	4.9	0.4	T3D2	13.1
Cage (polyetheretherketone)	3600	0.3	C3D4	—
Bone graft	450	0.25	C3D4	—

### Development of surgical models

In this study, the C5-C6 segment was chosen to simulate ACDF surgery because this segment is most prone to intervertebral disc degeneration. Firstly, the intervertebral disc, anterior longitudinal ligament, and posterior longitudinal ligament of the C5-C6 segment were removed. Using Solidworks, three cages of different sizes were constructed with a depth of 14 mm, a height of 6 mm, and widths of 7 mm, 10 mm, and 14 mm, simplified as w7-cage, w10-cage, and w14-cage. These cage models were assembled with the processed cervical spine model, and the combination function in Solidworks was used for Boolean subtraction to remove the overlapping parts of the vertebrae and implants, achieving a complete fit between the vertebra and the cage. The cages were meshed with hexahedral elements and the contact between the cage and the vertebra was set as Tie. The Tie contact was employed to simulate the complete fusion state. As shown in Fig. 1.

### Boundary and loading conditions

The lower end of the C7 vertebra was completely fixed, and a torque of 1 Nm was applied to the upper end of the C3 vertebra to simulate flexion, extension, left lateral bending, right lateral bending, left rotation, and right rotation. Additionally, a downward force of 73.6 N was applied in the axial direction to simulate the weight of the human head. To validate the effectiveness of the cervical spine model, a comparison was made between the finite element model in this study and previous experimental models [23, 24].

## Results

### Validation of the intact cervical spine model

**Fig. 2:**
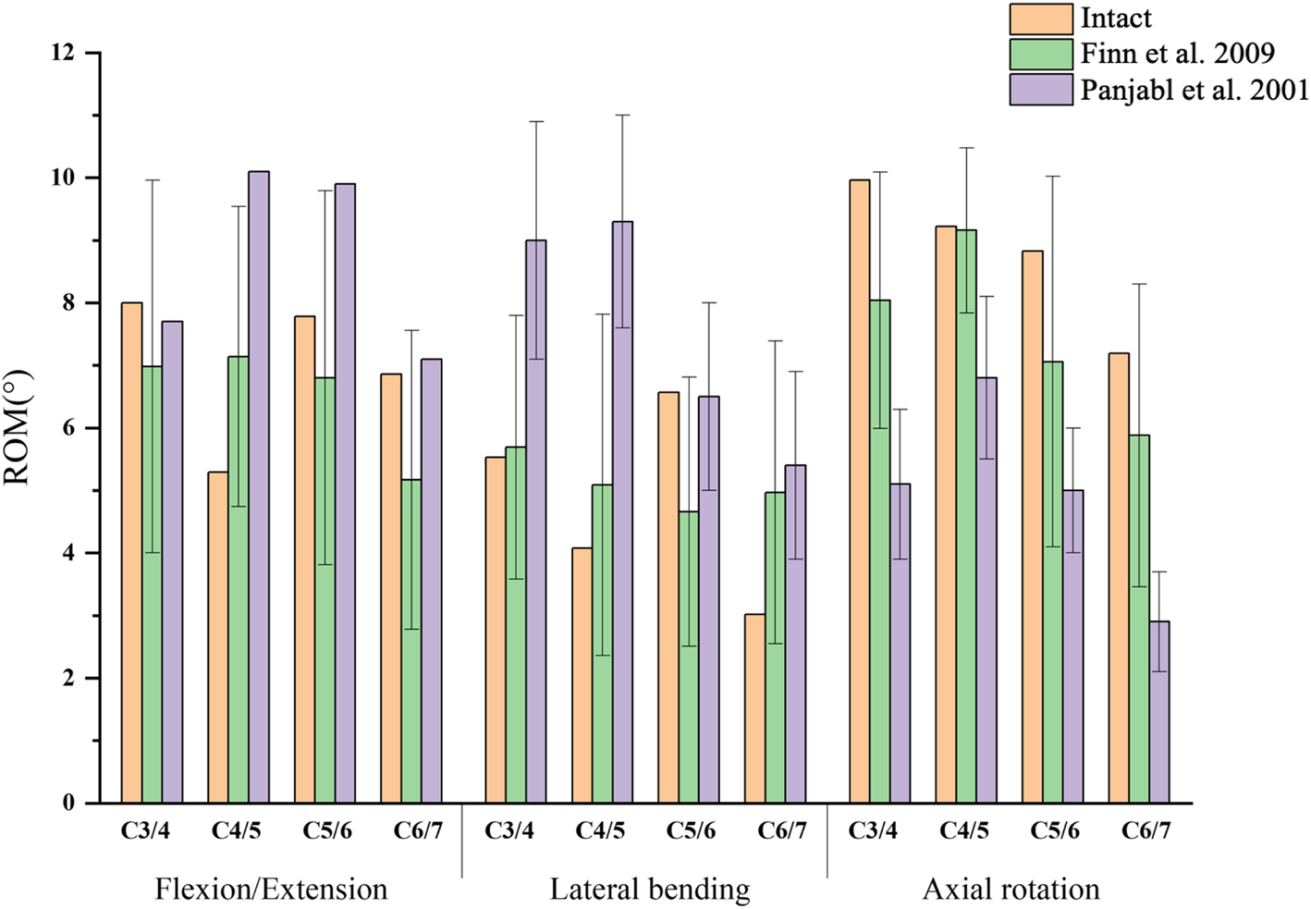
Validation of the intact C3-C7 finite element model

The comparison of the ROM between the intact model and the previous experimental models is shown in Fig. 2. The ROM for the C3/4, C4/5, C5/6, C6/7 segments in flexion–extension in the intact model was 8°, 5.29°, 7.78°, and 6.86° respectively. In lateral bending, the ROM was 5.53°, 4.08°, 6.57°, and 3.02°. In rotation, the ROM was 9.96°, 9.22°, 8.83°, and 7.18°, consistent with the previous model’s ROM. Therefore, the cervical spine finite element model in this study is validated as effective.

### Range of motion

**Fig. 3:**
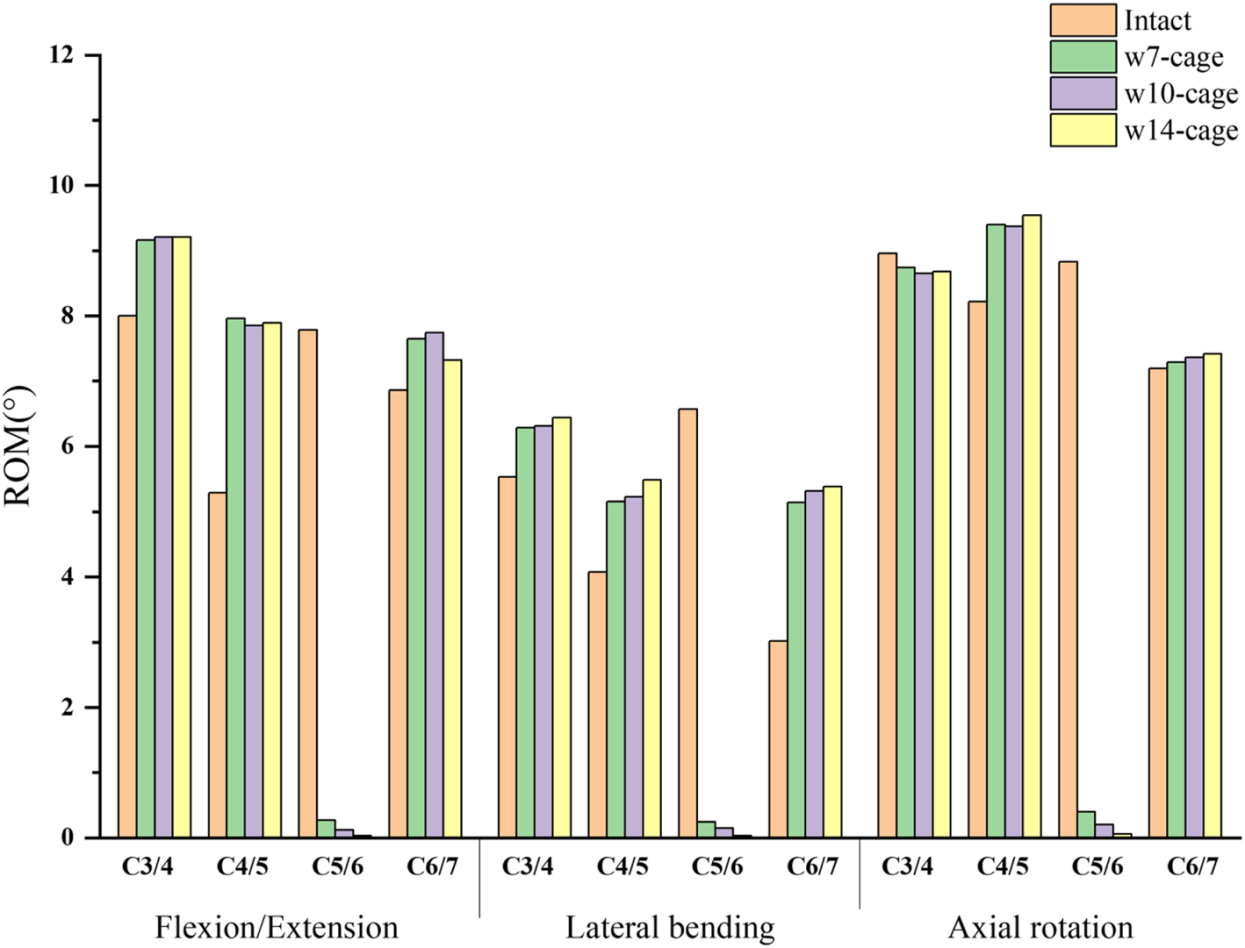
The ROM of C3-C4, C4-C5, C5-C6 and C6-C7 in the intact model and surgical models

The range of motion (ROM) for surgical segments and adjacent segments in different models is shown in Fig. 3. Relative to the intact model, the ROM in cervical surgical segments postoperatively experienced substantial reductions across all movements: for the w7-cage, there was a 96.5% reduction in flexion/extension, 96.3% in lateral bending, and 95.5% in rotation; for the w10-cage, reductions were 98.4% in flexion/extension, 97.7% in both lateral bending and rotation; and for the w14-cage, there was a 99.7% decrease in flexion/extension, 99.5% in lateral bending, and 99.3% in rotation. The ROM in the segments adjacent to the surgical segment showed a compensatory increase under all conditions. Compared to the intact model, the postoperative mobility in the surgical segments of all three surgical models was essentially lost. The ROM in the non-surgical segments did not show a significant change with the variation in the size of the cage.

### Intervertebral disc pressure

**Fig. 4:**
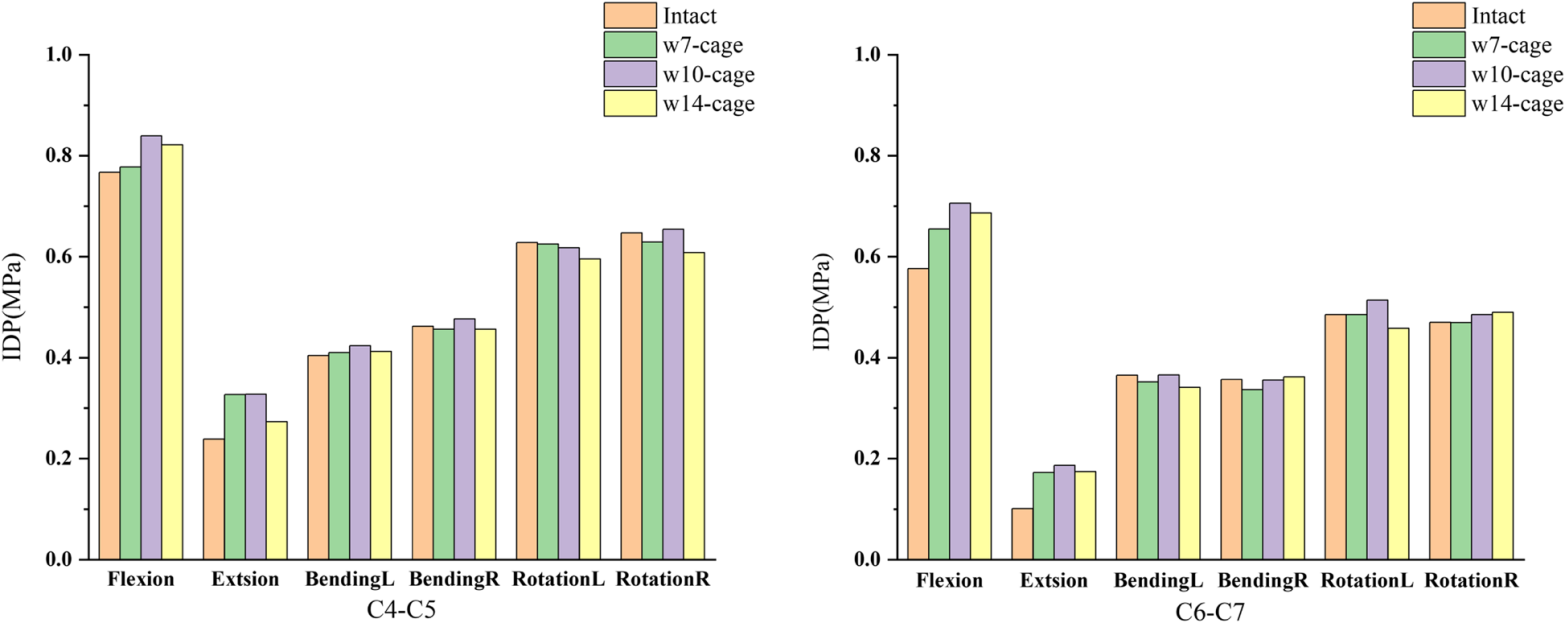
The IDP of C4-C5, C6-C7 in the intact and surgical models

**Fig. 5:**
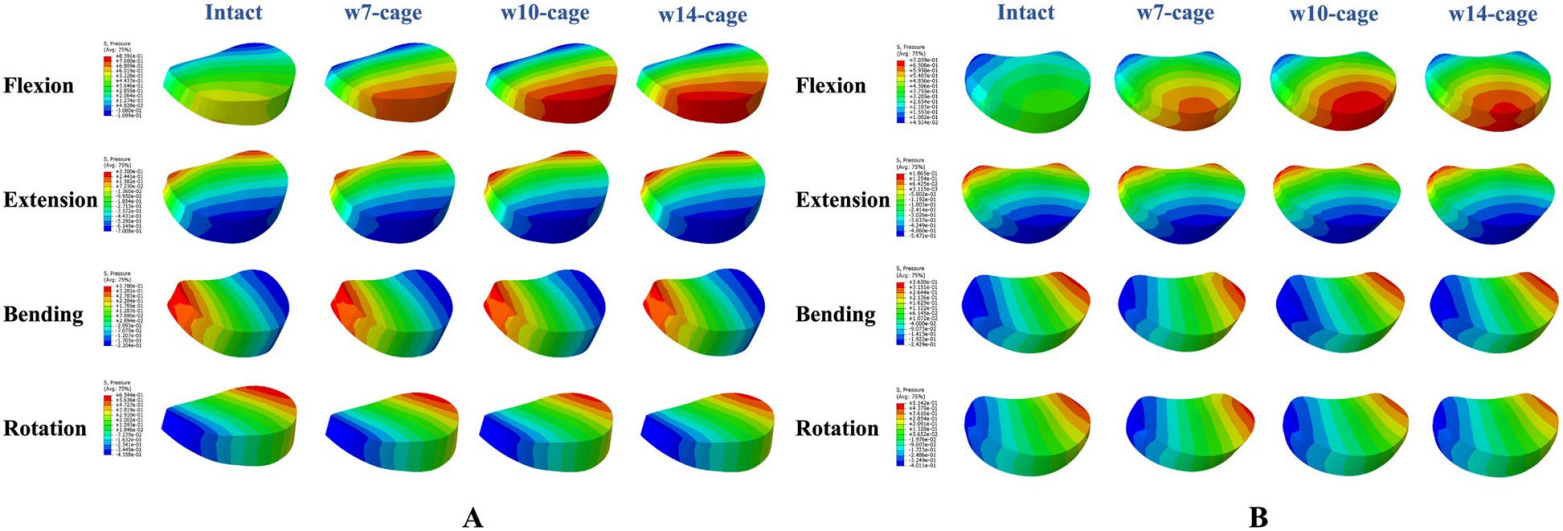
The intradiscal pressures distribution diagram of adjacent intervertebral discs. **A** C4/C5; **B** C6/C7

The changes in intervertebral disc pressure (IDP) in adjacent segments for different surgical models are shown in Figs. 4 and 5. For the upper adjacent segment (C4-C5), the maximum IDP value (0.8391 MPa) occurred in the w10-cage model during flexion, and the minimum value (0.2728 MPa) occurred in the w14-cage model during extension. Compared to the intact model, there was a noticeable increase in IDP during flexion and extension in the postoperative models, with the w7-cage model showing increases of 1.4% and 36.9%, respectively. The w10-cage model showed increases of 9.4% and 37.1%, respectively. The w14-cage model showed increases of 7.2% and 14.3%, respectively.

For the lower adjacent segment (C6-C7), the maximum IDP value (0.7059 MPa) was observed in the w10-cage model during flexion, and the minimum value (0.1723 MPa) in the w7-cage model during extension. Compared to the intact model, the w7-cage model showed an increase of 13.7% and 70.2% in flexion and extension, respectively. The w10-cage model showed increases of 22.5% and 84.3%, respectively. The w14-cage model showed increases of 19.2% and 71.9%, respectively. In lateral bending and rotation conditions, the three different sized cages did not have a significant impact on the IDP of the adjacent segments.

### Facet joint pressure

**Fig. 6:**
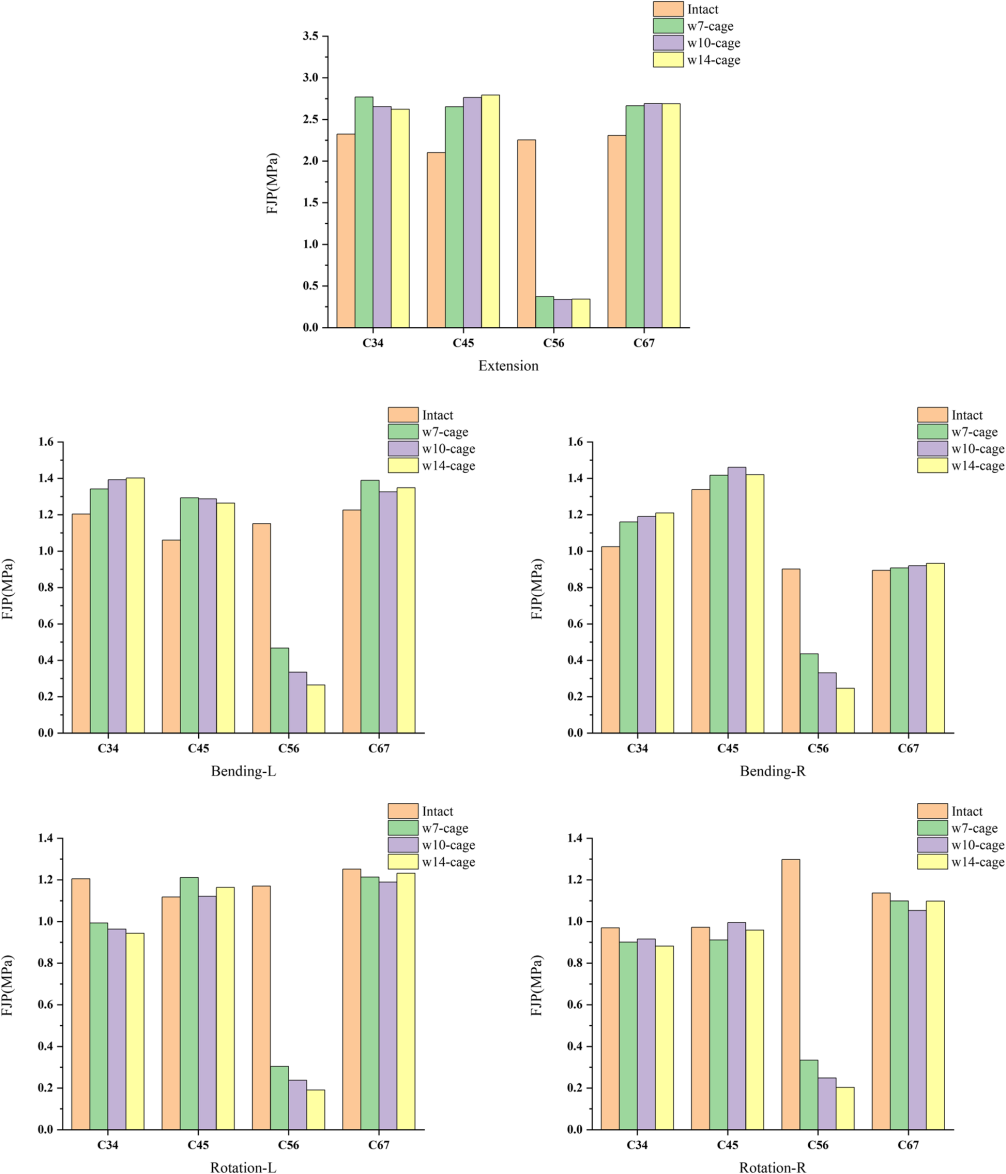
The FJP of C3-C4, C4-C5, C5-C6 and C6-C7 in the intact model and surgical models

In different postures, the predicted facet joint pressures (FJPs) from all adjacent segments with various models are shown in Fig. 6. The maximum FJP occurred in the w7-cage C3-C4 segment during extension, at 2.793 MPa. Compared to the intact model, the FJP in the surgical segments of all three postoperative models showed a decrease in all conditions relative to the intact model. In the surgical segment C5-C6, the average decrease in FJP for the three surgical models in extension, left lateral bending, right lateral bending, left rotation, and right rotation conditions was 84.47%, 69.14%, 62.55%, 79.15%, and 79.8%, respectively. The FJP in non-surgical segments showed varying degrees of compensatory increase under different conditions. With the decrease in the size of the cage, the FJP in the surgical segments gradually increased in all conditions except for extension. In the extension condition, the FJP in the surgical segments of all three surgical models was similar.

### Maximum stress in the endplate–cage interface at the treatment level

**Fig. 7:**
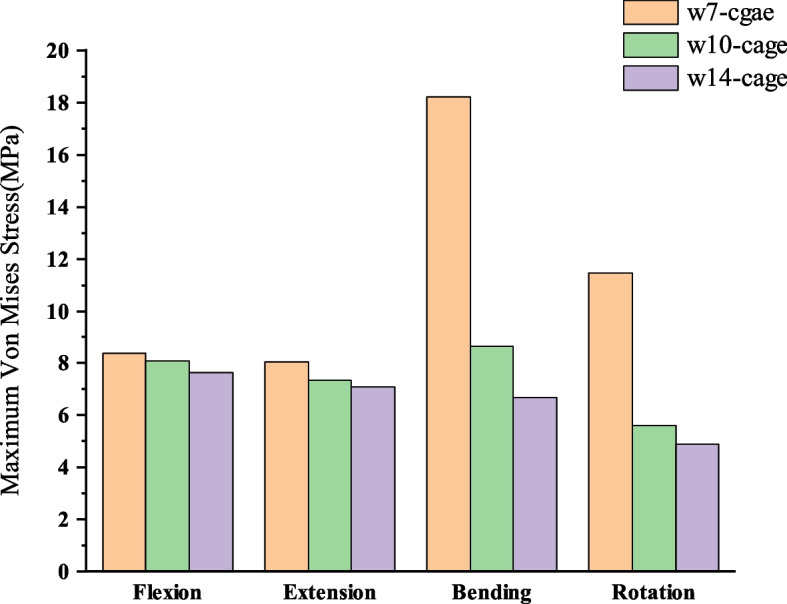
Maximum stress in the endplate–cage interface at the treatment level

The maximum von Mises stress on the endplate-cage contact surfaces for the three models is shown in Fig. 7. As the size of the cage decreases, the pressure on the endplate-cage contact surfaces increases under various conditions. In the lateral bending condition, compared to the w14-cage, the maximum stress on the w10-cage endplate contact surface increased by 14%, and on the w7-cage it increased by 234%. In the rotation condition, the maximum stress on the w10-cage endplate contact surface increased by 27%, and on the w7-cage it increased by 180%.

### Cage stress

**Fig. 8:**
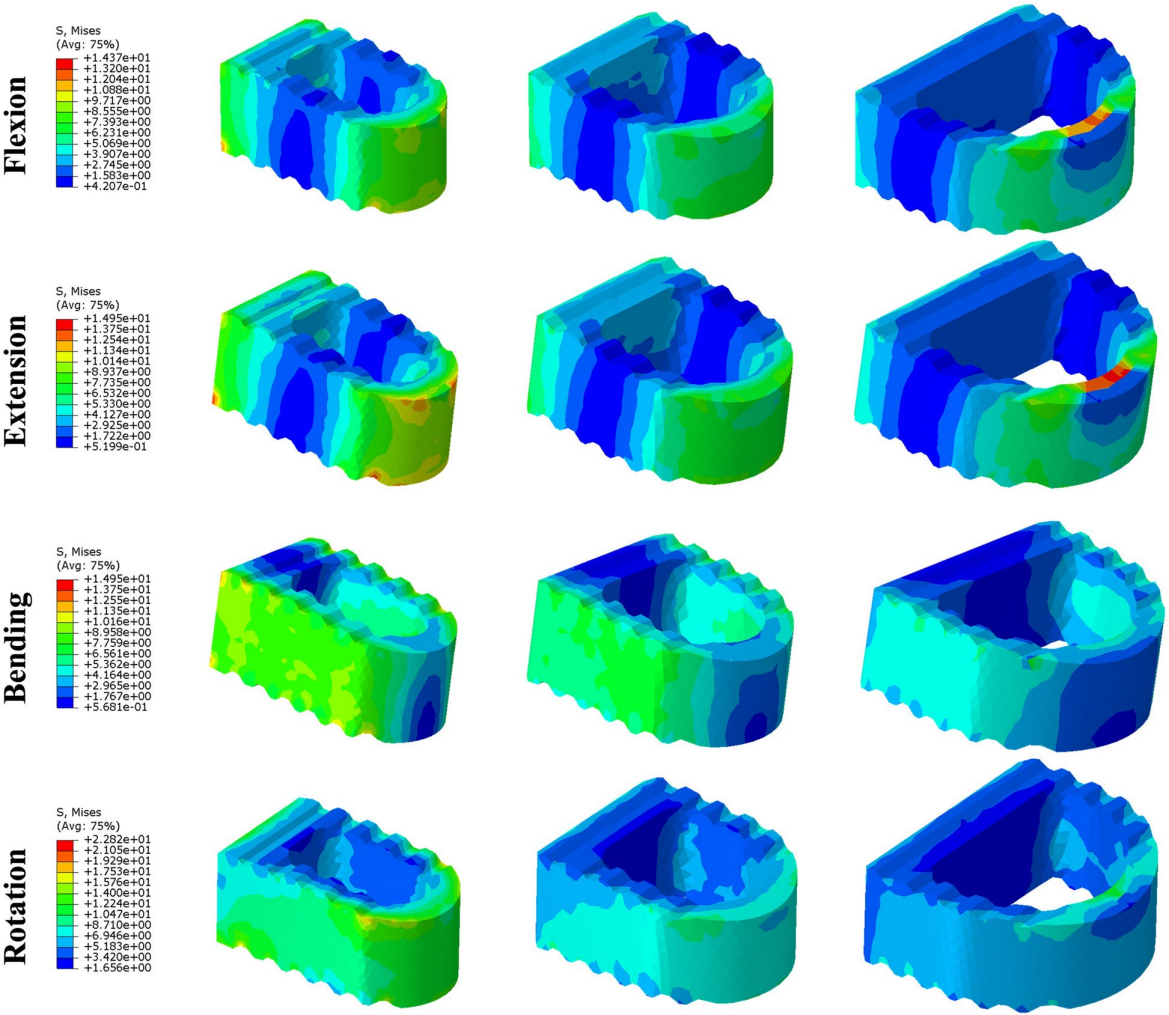
The stress distribution on the cage (w7-cage *VS.* w10-cage *VS.* w14-cage) at flexion, extension, bending and rotation

Stress contour diagrams in four motion conditions at surgical groups are shown in Fig. 8. As the size of the cage decreases, the von Mises stress on the cage increases in various conditions. During left and right rotation conditions, the maximum stress is concentrated on the upper surface of the cage. The maximum stress on the w7-cage surface is 12.42 MPa and 12.9 MPa, respectively, while on the w10-cage it is 6.619 MPa and 6.587 MPa, and on the w14-cage it is 4.822 MPa and 4.101 MPa, with the w7-cage showing the largest increase in pressure. In lateral bending conditions, the maximum stress is concentrated on both sides of the cage, ranging from 4.536 to 10.94 MPa, with the w7-cage experiencing the highest stress. During flexion and extension conditions, the maximum stress is concentrated on the anterior side of the cage, ranging from 4.283 to 10.84 MPa, with the w7-cage experiencing the highest stress.

## Discussion

This article provides a comprehensive analysis of postoperative subsidence risk, changes in vertebral stability, and potential postoperative complications. This is achieved by comparing ROM, IDP, FJP, cage stress, and endplate-cage interface stress in postoperative models with cages of varying sizes.

### Cervical stability

According to White and Panjabi [25, 26], cervical stability can be defined as the ability of the cervical spine to protect neuronal elements and maintain its integrity under physiological condition. ROM is an important parameter commonly used to evaluate vertebral stability in clinical, in vitro experiments, and FE simulations [27–29]. In this study, the postoperative cervical spine model of the C5-C6 segment showed a reduction in ROM by more than 95% under all conditions, indicating an almost complete loss of mobility in the surgical segment, consistent with previous clinical and experimental results [30, 31]. This is due to the implantation and fixation of the cage, which increases the rigidity of the intervertebral connection in the surgical segment, reducing its mobility. This rigid fixation provides a stable mechanical environment for bone growth, preventing the formation of pseudarthrosis due to excessive relative movement between the bone graft and the bone graft bed [32].

As the size of the cage decreases, there is a trend of increasing ROM in the surgical segment. In rotation, the maximum difference in ROM between the w7-cage and w10-cage is 0.36 degrees, suggesting that a reduction in cage size may slightly decrease the stability of the surgical segment. Considering that this study was conducted in a state of complete fusion, smaller cages may face more severe instability in the early stage of implantation. Therefore, improving the stability of small cages in the early stage of the surgical phase is particularly important. In the early stage of bone healing, interbody fixation is initially used as a load-bearing element to create a stable biomechanical environment [33, 34]. The method of fixation of the cage in the early implantation period plays a crucial role in postoperative vertebral stability. The cage-plate fixation method is a completely rigid fixation, which maintains the intervertebral height and stability of the surgical segment by fixing the upper and lower vertebrae with a plate [34]. However, due to the large volume of the anterior plate, it cannot be entered through the endoscopic surgical approach. The Zero-P cage, without using an anterior plate and only fixed with screws or VerteBRIDGE self-locking plates, reduces the volume of the implantation system. Additionally, it provides good immediate stability after implantation, making it very suitable as a fixation method for ACDF surgery under an endoscopic channel [35–37]. Overall, reducing the size of the cage may increase the risk of stability decrease during the surgical phase, leading to the formation of pseudarthrosis. To reduce this risk, it is recommended to choose a zero-P cage to enhance the stability of the fixation.

### Risks of degeneration at adjacent segments

Adjacent segment disc degeneration is a common complication after ACDF surgery. In the study by Iampreechakul et al. [38], the incidence of adjacent segment pathology was 13.6% and 25.6%, respectively. Many studies have shown that the compensatory increase in ROM of non-fused motion segments in patients after ACDF surgery may elevate IDP, leading to segmental degeneration [39]. Eck et al. [40] found that after single-segment fusion at the C5/6 segment, there was a significant increase in mobility and IDP in the adjacent upper and lower segments. The compensatory increase in mobility of non-fused segments may lead to an increase in FJF, which is closely related to the exacerbation of segmental degeneration. In vitro experiments have also confirmed an increase in FJFs in adjacent segments after ACDF fusion, which could be one of the factors accelerating Adjacent Segment Degeneration (ASD) [41]. Therefore, changes in IDP and FJP in postoperative adjacent segments are important indicators for assessing ASD. In this study, the postoperative IDP and FJP in adjacent segments of the three models increased compared to preoperative values, consistent with previous experimental results. There were no significant differences in postoperative IDP and FJP among the three models, which is consistent with their similar ROM results, indicating that under a state of complete fusion, small-sized cages do not cause postoperative instability or increase the incidence of ASD compared to normal-sized cages.

### Subsidence

Cage subsidence is a common failure mode of interbody cages. Short-term cage subsidence can lead to changes in the local alignment of the cervical spine [34]. Previous studies have shown that the material, geometric shape, and fusion level of the cage are potential risk factors for cage subsidence [20, 42, 43]. In this study, the three cages were of the same material and shape, differing only in size. Some evidence suggests that subsidence is more common in smaller cages, possibly indicating that a larger contact area reduces the risk of cage subsidence [43, 44]. However, some researchers have found that oversized cages also have a higher subsidence rate [38].

Suh et al. [20] pointed out that the contact strength between the implant and vertebral body is a major factor affecting subsidence, which depends on the mechanical properties of the material of the cage and the vertebral bone part in contact with the cage. In this study, the w7-cage and w10-cage, due to reduced width, showed decreased support for the sides of the vertebrae, resulting in a significant increase in stress on the cage-endplate contact surface under lateral bending and rotation conditions. Particularly with the w7-cage, there was a marked stress concentration, resulting in the maximum stress on the contact surface being almost twice as high as that of the w14-cage. While the w10-cage increased the maximum stress on the contact surface by only 10% to 30% compared to the w14-cage. Thus, decreasing the cage size escalates the stress on the endplate-cage contact area, consequently elevating the risk of subsidence. Compared to a regular-sized cage, the risk of subsidence caused by the w7-cage is significantly higher.

From the stress distribution diagrams, it can be seen that the w7-cage has severe stress concentration on its surface, while the w10-cage has a relatively uniform stress distribution. According to Wolff’s Law, previous studies have reported that excessive concentration of stress on implants can limit the bone growth in a low-stress state leading to a reduction in bone mass. Excessive stress concentration can cause a stress shielding effect, leading to bone resorption and implant loosening, ultimately causing implant subsidence [45].

Besides size, the material of the cage also plays a crucial role in the contact strength between the cage and the vertebral body. Cages made of metal materials have a higher modulus of elasticity, leading to significantly higher stress at the contact surface with the vertebral body compared to non-metal cages. PEEK material, with an elastic modulus similar to that of cancellous bone, can effectively reduce the stress on the endplate-cage contact surface [46, 47]. In this study, even the stress on the contact surface of the w7-cage was far less than that of commercially available titanium alloy cages. Using PEEK material for small-sized cages can effectively mitigate the increase in stress caused by size reduction, thereby reducing the risk of subsidence.

This study presents several limitations that should be considered. First, the employed cervical spine finite element model was developed based on the anatomical data of a single individual, which means it primarily reflects trends in the cervical spine’s mechanical response under varied loading conditions, rather than providing a universal representation. Second, while finite element analysis serves as a valuable computational tool for modeling, it falls short of accurately replicating the complex process of bone fusion. Third, the analysis conducted in this study is predicated on the assumption of complete fusion, focusing on how cage size influences the postoperative biomechanics of the vertebrae under this specific condition. Finally, the model lacks the incorporation of muscular structures, instead opting for an axial load to simulate the combined effects of head weight and muscular forces. These factors highlight the need for cautious interpretation of the study’s findings and suggest areas for future research enhancement.

## Conclusions

The results indicate that under a state of complete fusion, changes in the size of the cage do not significantly impact vertebral stability, nor do they increase the risk of adjacent segment disc degeneration. However, they do have a significant effect on the stress distribution between the cage and the endplate. The use of a w7-cage leads to a substantial increase in maximum stress on the cage-endplate contact surface, and the stress distribution on the surface of the cage is noticeably concentrated, thereby elevating the risk of cage subsidence. In comparison, the w10-cage shows a smaller increase in maximum stress on the cage-endplate contact surface compared to the normal-sized cage, and the stress distribution on the cage is more uniform. Using PEEK as the cage material can effectively reduce the stress on the cage surface, thus reducing the risk of subsidence. In summary, a 10 mm wide PEEK cage has a smaller impact on the postoperative biomechanical performance of the vertebrae compared to a normal-sized cage, making it a viable option for ACDF via an endoscopic surgical approach.

## Data Availability

No datasets were generated or analysed during the current study.

## Abbreviations

ACDF: Anterior cervical discectomy and fusion
PECD: Percutaneous endoscopic cervical discectomy
CT: Computer tomography
ALL: Anterior longitudinal ligament
PLL: Posterior longitudinal ligament
LF: Ligamentum flavum
CL: Capsular ligament
IL: Interspinous ligament
ROM: Rang of motion
IDP: Intervertebral disc pressure
FJP: Facet joint pressures
FJF: Facet joint force
FE: Finite element
ASD: Adjacent segment degeneration
